# Track-Before-Detect Framework-Based Vehicle Monocular Vision Sensors

**DOI:** 10.3390/s19030560

**Published:** 2019-01-29

**Authors:** Hernan Gonzalez, Sergio Rodriguez, Abdelhafid Elouardi

**Affiliations:** Laboratory SATIE (Systèmes et Applications des Technologies de l’Information et de l’Energie), CNRS (UMR 8029), Université Paris Sud, 91405 Orsay, France; sergio.rodriguez@u-psud.fr (S.R.); abdelhafid.elouardi@u-psud.fr (A.E.)

**Keywords:** motion segmentation, monocular camera, structure from motion, embedded systems

## Abstract

This paper proposes a Track-before-Detect framework for a multibody motion segmentation (named TbD-SfM). Our contribution relies on a tightly coupled tracking before detection strategy intended to reduce the complexity of existing Multibody Structure from Motion approaches. Efforts were done towards an algorithm variant closer and aimed to a further embedded implementation for dynamic scene analysis while enhancing processing time performances. This generic motion segmentation approach can be transposed to several transportation sensor systems since no constraints are considered on segmented motions (6-DOF model). The tracking scheme is analyzed and its performance is evaluated under thorough experimental conditions including full-scale driving scenarios from known and available datasets. Results on challenging scenarios including the presence of multiple and simultaneous moving objects observed from a moving camera are reported and discussed.

## 1. Introduction

The increasing introduction of Autonomous Vehicles (AV) and Advanced Driver Assistance Systems (ADAS) into the marketplace is essential in the design of Intelligent Transportation Systems (ITS). Recently, these areas have shown an active development towards unmanned transportation solutions (Car autonomy SAE Level 4). In this context, perception is a critical task since it provides meaningful, complete and reliable information about the vehicle surroundings [1,2]. Several studies have demonstrated that vision perception is an essential sensing method for scene analysis [3,4,5]. Vision-based techniques such as Visual Simultaneous Localization And Mapping (VSLAM) are well-suited for inferring ego-localization by reconstructing simultaneously the environment structure [6]. Another well-known technique considered for monocular vision applications is Structure-from-Motion (SfM). This method estimates the camera pose from the image motion and the 3D structure of the scene, up to a scale factor. In this paper a Track-before-Detect framework coupled to a multibody SfM (TbD-SfM) methodology is deployed to detect and to segment multiple motions in dynamic scenes. In the first stage, our algorithm is initialized using the motion segmentation approach described in [7]. The initialization procedure provides a rough feature segmentation of static feature points (ego-motion) and dynamic feature points (euro-motions). Further, the euro-motions are tracked by the use of a bank of Bayesian filters so as to observe and predict the image position of these objects in next frames. Then, the feature points inside of the tracked areas are refined to precisely estimate the euro-motions. The remaining feature points are used to compute the ego-motion. A robust formulation based on RANSAC is proposed for finding the motion hypotheses in each tracked area. Finally, the motions are computed using SfM formulation [8].

### 1.1. Related Works

Image motion segmentation has been widely studied using different approaches as it is surveyed in [9]. Tomasi and Kanade [10] presented a well-known factorization approach that became very popular due to its simplicity for recovering scene geometry and camera motion. Later in [8], a factorization framework of multibody SfM was proposed. This approach considers a static camera that observes a scene with moving objects. A common drawback of all these approaches is their sensibility to noise conditions.

Vidal et al. [11] proposed the use of an algebraic and geometric method for estimating 3D motion and segmenting multiple rigid-body motions from two perspective views. The method relies on multibody epipolar constraint and its corresponding multibody fundamental matrix. The complexity of such an approach is unbounded since the amount of required image pairs grows quartically in presence of more than two simultaneous motions. Goh and Vidal [12] proposed the Locally Linear Manifold Clustering (LLMC). It consists on a nonlinear dimensionality reduction which finds different clusters where feature points are segmented. This unsupervised method does not require any prior knowledge but the clusters results are not consistent. Alternatively, Vidal and Hartley [13] addressed the multiple rigid-body motion segmentation using a three view geometry model. In detail, a multibody trifocal tensor encodes the parameters of all rigid motions and transfers epipolar points and lines between pairs of views. This information is used to obtain an initial clustering. Trifocal tensors and motion segmentation are then refined.

Li et al. [14] proposed an extension of the iterative Sturm/Triggs (ST) algorithm to alternate between the depth estimation and the trajectories segmentation. Then, a Generalized Principal Component Analysis (GPCA) or a Local Sub-space Affinity (LSA) is performed for data clustering in multiple linear subspace. The method reduces the processing time, however, it does not improve the motion segmentation error.

Ozden et al. [15] applied the multibody SfM formulation to compute the 3D structure of objects and the camera motion via geometry decomposition using the five-points algorithm. The approach uses three non-consecutive frames of the sequence for segmenting (the first, middle and last frame of the sequence) in order to obtain stable results. Rao et al. [16] suggested a subspace separation method based on expectation-maximization and spectral clustering named Agglomerative Lossy Compression (ALC). This non-iterative algorithm applies the principles of data compression and sparse representation to the motion segmentation. Zapella et al. [17] proposed a solution based on a bi-linear optimization procedure to refine a initial segmentation following metric constraints and the sparsity matrix of the 3D shape of moving objects.

Dragon et al. [18] suggested the multi-scale clustering (MSCM). This method is performs top-down split and merge for segmenting between two consecutive frames. Image segments are then split until they are consistent and finally merged to neighboring segments until convergence. MSCM combines frame-to-frame motion segmentation in a time-consistent manner. In [19] was implemented the Discrete Cosine Transform (DCT) to segment motion. To this end, a non-linear optimization scheme decomposes the input trajectories into a set of DCT vectors. Then, a spectral clustering technique is used to separate the foreground trajectories from the background trajectory. Jung et al. [20] studied a randomized voting (RV) method. The algorithm is based on epipolar constraints and Sampson distances between feature points and theirs epipolar line. The motions that are correctly estimated get high scores and invalid motions get low scores. The score is used to separate the motions in clusters. Li et al. [21] presented a subspace clustering approach called Mixture of Gaussian Regression (MoG Regression), which employs the MoG model to characterize noise with a complex distribution. Then, it is applied a clustering method based on the spectral clustering theory. Tourani et al. [22] carried out the hypothesis generation using the RANSAC procedure. An over-segmentation is implemented by a long-term gestalt-inspired motion similarity constraints, into a multi-label Markov Random Field (MRF). Segmented Motions are merged in clusters based on a new motion coherence constraint named in-frame shear. Sako et al. [23] proposed to segment motions by hierarchically separating trajectories into 2D and 3D affine spaces. The affine space is determined by the rank value of the trajectory matrix and computed by using the Minimum Description Length (MDL). Then, the average likelihood of the identified trajectories is computed and those associated to large likelihood are segmented again. Zhu et al. [24] suggested a general multilayer framework to detect dynamic objects based on motion, appearance and probability. The motion is estimated with Gaussian Belief Propagation and employed for propagating the appearance models and the prior probability. Kernel Density Estimation is applied to obtain the probability map as output. Recently, [7] introduces an iterative approach for robust estimations of multiple structures and motions from perspective views. This work was then extended in [25] by introducing kinematic constrains of ground vehicles in order to reduce the mathematical complexity of the motion-estimation procedure.

### 1.2. Contributions

The main contributions of our work are summarized below:

- A novel tracking framework for general 6-DOF simultaneous motion segmentation based on temporal filtering and RANSAC formulation. This monocular vision sensor approach minimizes the amount of hypothesis to achieve a good motion segmentation without any prior knowledge about observed motions.
- A thorough experimental procedure is reported on full-scale dynamic scenarios. Based on the obtained results, our method improves the motion detection on challenging dynamic scenes without need of a fine tuning procedure.
- A comparison with other state-of-the-art techniques is provided in terms of segmentation and reprojection errors as proposed in [18,19,20,21,22,23,24,25,26,27,28,29,30,31,32]. The outliers ratio is also provided as an indicator of the number of segmented feature points.

### 1.3. Paper Outline

This paper is structured as follows: Section 2 is devoted to introduce the theoretical concepts of single and multiple motion formulation of the SfM factorization approach. Section 3 explains the methodology fundamentals for multibody motion segmentation using SfM. In Section 4, the proposed framework is detailed with a particular focus on the strategy for reducing the number of hypotheses required for the multibody motion segmentation. Finally, Section 5 presents the experimental protocol and the evaluation of the obtained results under full scale dynamic scenes.

## 2. Structure from Motion Factorization

### 2.1. Single Motion Formulation

Let us consider an object as a rigid body and its motion to be represented and sampled by image feature points. From the viewpoint of a moving camera, the feature points observed on a scene can lie on static and dynamic objects. Under these assumptions, the factorization approach in [10] considers a group of 2D feature points to be tracked and matched over *f* consecutive frames in a sequence of images. The cardinality of this set of points is denoted *p*. Based on these observations two problems are addressed: (i) recovering the unknown 3D scene structure up to a scale factor and (ii) estimating ego-camera motion.

A static scenario observed from a moving camera constitutes the simplest use-case. Let us consider $W\in R^{3f\times p}$ as the measurement matrix composed of the image coordinates of the feature points along the sequence. Each column vector of this matrix represents the feature point position by frame as $w_{p}={\left[w_{1p},w_{2p},\ldots ,w_{\mathrm{fp}}\right]}^{T}$ with $w_{\mathrm{fp}}\in R{}^{3f\times 1}$. The camera motion between frames is modeled by a rigid transformation, $M=\left[R|t\right]$, where $M\in R{}^{3f\times 4}$, $R\in R^{3\times 3}$ and $t\in R^{3\times 1}$ stands for rotation and translation respectively. Finally, $S\in R^{4\times p}$ is the structure composed of 3D homogeneous coordinates of the feature points $s_{p}={\left[s_{x},s_{y},s_{z},1\right]}^{T}$ as stated in Equation (1):

(1) $$\begin{array}{c} W=\left[\begin{array}{cccc} w_{11} & w_{12} & \cdots & w_{1p}\\ w_{21} & w_{22} & \cdots & w_{2p}\\ \vdots & \vdots & \vdots & \vdots \\ w_{f1} & w_{f2} & \cdots & w_{fp} \end{array}\right],\;M=\left[\begin{array}{c} M_{1}\\ M_{2}\\ \vdots \\ M_{f} \end{array}\right]\\ S=\left[\begin{array}{cccc} s_{1} & s_{2} & \cdots & s_{p} \end{array}\right] \end{array}$$

Thus, the single motion general formulation of SfM is as follows:

(2) $$W_{3f\times p}=M_{3f\times 4}\cdot S_{4\times p}$$

The bilinear elements *M* and *S* are computed by factorizing *W*. The solution to the Equation (2), namely $\tilde{W}$, stands for the best rank-4 approximation to the matrix *W* given by the rank-4 estimates of motion $\left(\tilde{M}\right)$ and structure $\left(\tilde{S}\right)$ as:

(3) $${\tilde{W}}_{3f\times p}\approx {\tilde{M}}_{3f\times 4}\tilde{S}{}_{4\times p}$$

### 2.2. Multiple Motions Formulation

In a scene composed of multiple motions [8], multibody motion segmentation facilitates the computation of the camera motion and the structure of all the rigid bodies in the scene using the general formulation (see Equation (2)). The multibody trajectory matrix *W* is consisted of the trajectory matrix of the *n* independent motions, each of them are represented by $W_{n}\in R^{3f\times p}$. The multibody camera motion $M\in R{}^{3f\times 4n}$ is computed with respect to each *n* independent body motion and denoted as $M_{n}\in R{}^{3f\times 4}$. Finally, multibody 3D structure, $S\in R^{4n\times p}$, is built in a sparse shape enclosing the structure of each body, $S_{n}\in R^{4\times p}$, in a diagonal matrix. The general multibody SfM formulation is:

(4) $$\left[W_{1}\left|\ldots \right|W_{n}\right]=\left[M_{1}\left|\ldots \right|M_{n}\right]\cdot \left[\begin{array}{ccc} S_{1} & 0 & 0\\ \vdots & \ddots & \vdots \\ 0 & 0 & S_{n} \end{array}\right]$$

Equation (4) is solved by factorizing each motion individually.

## 3. Scene Motion Segmentation Methodology

The SfM procedure stated in [7] is considered in this study to detect motion and to recover trajectories from multiple views. Let us refer to this method as baseline method. This methodology is applied to scenes composed of static and dynamic objects. Hereafter, we consider monocular image sequences captured on board a moving vehicle. Images are analyzed and processed through a temporal sliding window and feature points are extracted.

The detection process starts by randomly sampling a feature points set of two consecutive frames from the trajectory matrix. These points are employed to recover the relative motion between the frames (M) and the structure (S). This stage is carried out on the same set of feature points along a temporal sliding window of size Γ, so as to retrieve a trajectory which minimizes the reprojection error.

A new motion hypothesis, $\left(W_{n}^{hyp}\right)$, is instantiated from any set of features achieving a reprojection error less than a threshold. A motion hypothesis is defined as a possible trajectory matrix that satisfies the reprojection error criterion and represents the *n*th motion of the observed scene. Since the number of observed motions is unknown, new trajectories are built until all feature points are assigned. At the end of this procedure, the scene segmentation is then composed of *n* motions. As a result, the best scene segmentation in terms of reprojection errors is selected.

In the remaining of this section, it is detailed how to determine the number of sampling trials that are required to instantiate a new motion hypothesis. Next, the hypotheses evaluation method is introduced and the association criterion of a feature point to a motion hypothesis is detailed.

### 3.1. Recover Motion and Structure

The trajectory matrix *W* is normalized using the 8-point algorithm and represented by $\bar{W}$. A set of *k* points in two consecutive frames are sampled from the matrix $\bar{W}$ and defined by ${\bar{w}}_{f}={\left[p{}_{1},p_{2},\ldots ,p{}_{k}\right]}^{T}$ and its consecutive frame ${\bar{w}}_{f}^{\prime }={\left[p_{1}^{{}^{\prime }},p_{2}^{{}^{\prime }},\ldots ,p_{k}^{{}^{\prime }}\right]}^{T}$. A feature point ${\bar{p}}_{i}$ is selected randomly [33] and ${\bar{p}}_{i}^{\prime }$ features are associated following a nearest neighbor criterion with a probability distribution modeled by Equation (5). The values of ζ and ρ are selected heuristically in function of the probability scale.

(5) $$P\left({\bar{p}}_{i}|{\bar{p}}_{i}^{\prime }\right)=\left\{\begin{array}{cc} \frac{1}{\zeta }exp-\frac{∥{\bar{p}}_{i}-{\bar{p}}_{i}^{\prime }{∥}^{2}}{\rho^{2}} & if\;{\bar{p}}_{i}\neq {\bar{p}}_{i}^{\prime }\\ 0 & if\;{\bar{p}}_{i}={\bar{p}}_{i}^{\prime } \end{array}\right.$$

The vectors are used to enforce epipolar constraints over the matrix *E* as it is written in Equation (6). *E* is computed in a least square form, Ax=0, where *A* are the coefficients of ${\bar{w}}_{f}$ and ${\bar{w}}_{f}^{\prime }$, and *x* the essential matrix *E*.

(6) $${\bar{w}}_{f}^{\prime T}\cdot E\cdot {\bar{w}}_{f}=0$$

The motion is defined as $\tilde{M}=\left[R|t\right]$ where the rotation and translation are recovered by means of a singular-value decomposition (SVD) of the essential matrix *E* as:

(7) $$UDV^{T}=SVD\left(E\right)$$

The possible four solutions $\left[UQV^{T}\pm U_{3c}\right]$ and $\left[UQ^{T}V^{T}\pm U_{3c}\right]$ are evaluated in order to select the only valid combination. Finally, the structure ${\tilde{S}}_{k}\in R^{4\times k}$ is estimated with a SVD of the camera projection matrix of two consecutive images.

### 3.2. Generation of Motion Hypotheses

A motion hypothesis is estimated from the motion $\tilde{M}$ and the Structure ${\tilde{S}}_{k}$ recovered using the vectors ${\bar{w}}_{f}$ and ${\bar{w}}_{f}^{\prime }$, (see Section 3.1), in each consecutive pair of frames along the sliding window. The matrix ${\tilde{\bar{W}}}_{k}$ is determined by the Equation (2) for each sampling trial. The reprojection error is evaluated for each pair of frames and accumulated in the sliding window. A hypothesis is accepted if the reprojection error on the sliding window is less than a threshold $\epsilon_{hyp}$, such as:

(8) $$\sum_{f=1}^{\Gamma }∥W_{k}-\left(\tilde{M}\cdot {\tilde{S}}_{k}\right)∥\leq \epsilon_{hyp}$$

If the hypothesis is validated, the trajectory matrix, the motion and the structure are kept in ${\tilde{W}}_{k}^{h}$, ${\tilde{M}}^{h}$ and ${\tilde{S}}_{k}^{h}$, respectively. If the hypothesis is discarded, a new set of *k* feature points are sampled until the number of sampling trials, ψ, is reached.

#### Association Criterion of a Feature Point and a Motion Hypothesis

Given the motion ${\tilde{M}}^{h}$ and the feature points matrix $\bar{W}$, the structure $\left({\tilde{S}}^{h}\right)$ is calculated using linear triangulation method [34]. The motion ${\tilde{M}}^{h}$ is applied to the structure ${\tilde{S}}^{h}$ in Equation (2) to obtain $\tilde{\bar{W}}$. The reprojection error is computed for each point in the sliding window as in Equation (9). Feature points achieving a reprojection error less than a threshold $\epsilon_{pto}$ are kept in the group $W_{n}$ and removed from *W*. The threshold $\epsilon_{pto}$ is defined as the maximum reprojection error allowed by feature point.

(9) $$∥W-\tilde{W}∥\leq \epsilon_{pto}$$

Finally, the structure $S_{n}$ is updated using the feature points satisfying the reprojection error criterion $\left(W_{n}\right)$ and the motion ${\tilde{M}}^{h}$. Motion hypotheses are created from the remaining points $\left(W-W_{n}\right)$ until all the trajectory points in *W* are assigned or rejected as outliers.

### 3.3. Sampling Trials for Motion Segmentation

The motion segmentation addressed in this paper is a probabilistic procedure. This procedure is carried out iteratively on the set of features until all observed motions are detected. It is necessary then to determine the number of sampling trials (ψ) required to achieve good results with a probability $p_{r}$. ψ is estimated relying on the RANSAC formulation, where ϵ stands for the probability that any selected data point is an outlier, such as:

(10) $$\psi =\frac{\log\left(1-p_{r}\right)}{\log\left(1-{\left(1-\epsilon \right)}^{k}\right)}$$

It is worth to mention that this formulation leads to detect at first the dominant motion of the scene. This motion corresponds to that of the camera (i.e., ego-motion). In the subsequent iterations, motions from features lying on dynamic objects are detected.

### 3.4. Evaluation of a Motion Hypothesis

After ψ trials, multiple solutions for an observed motion can satisfy the condition stated in Equation (9). The solution with the smallest Euclidean distance between the trajectory matrix *W* and the hypotheses estimations $\left({\tilde{W}}_{n}\right)$ is selected as the best motion hypothesis. For the first motion (n=1), this is considered as the dominant motion since it retrieves the higher consensus of the features point set.

The outline of the motion segmentation process is summarized in the Algorithm 1.

**Algorithm 1** Motion Segmentation Algorithm
1: **procedure**
Segmentation(*W*)2:  k=8▹ minimum number of points3:  ψ▹ number of hypotheses4:  *n* = 0▹ counter of motions5:  **while**
hyp≤ψ
**do**6:   hyp=0▹ hypotheses counter7:   **while** number of feature points in (W)⩾k
**do**8:    **while** reprojection error $⩾\epsilon_{hyp}$
**do**9:     $\bar{W}=$ Normalize(W)10:     Sample *k* points from $\bar{W}$11:     Compute $\tilde{M}$ and ${\tilde{S}}_{k}$, Section 3.112:     Compute ${\tilde{\bar{W}}}_{k}$ with $\tilde{M}$ and ${\tilde{S}}_{k}$13:     Compute reprojection Error for the hyp14:    **end while**15:    n=n+116:    ${\tilde{M}}^{h}=\tilde{M}$, ${\tilde{S}}_{k}^{h}={\tilde{S}}_{k}$17:    Apply ${\tilde{M}}^{h}$ over the remaining feature points18:    Compute $\tilde{\bar{W}}$ with ${\tilde{M}}^{h}$ and ${\tilde{S}}^{h}$19:    Compute reprojection Error point20:    **if** reprojection Error point $w_{p}$
$\leq \epsilon_{pto}$
**then**21:     Add the points to $W_{n}$22:     Remove the points from *W*23:    **end if**24:   **end while**25:   hyp=hyp+126:  **end while**27:  **return**$W_{n},M_{n},S_{n}$▹ Trajectory matrix segmented, Motion and Structure28:
**end procedure**


## 4. Track-Before-Detect Framework

The multibody SfM based approach introduced by Sabzevari et al. [7] has proved to be suitable for achieving scene motion segmentation following a closed-form formalism. However, the computational complexity of this strategy is vast and it increases with the number of observed motions. To alleviate this limitation, the authors recently proposed in [25] a speeded up variant of the procedure taking advantage of motion model priors in context of a ground vehicle application. With lost of generality, the reformulated problem was limited to 2-DOF instead 6-DOF reducing drastically the complexity.

A Track-before-Detect-SfM(TbD-SfM) framework is proposed for improving scene motion segmentation by simultaneously detecting and tracking multiple dynamic image regions. This method is intended to efficiently limit the computational complexity without motion prior constraints on the scene dynamics. As a result, this method improves the inference of the observed motions number, deals with more complex scenarios including partial occlusions and preserves a high feature point density on tracked dynamic regions.

A drastic decrease on the sampling and the evaluation of scene motion hypotheses is achieved since dynamic regions are tracked and efficiently exploited to limit the solution exploration space.

The TbD-SfM framework needs to be initialized with a set of rough motion segments. To this end, factorization-based scene motion segmentation presented in Section 3 is employed. Alternatively, multiple-view motion detection can also be performed [35]. Based on the rough scene segmentation a multi-target tracking (MTT) is started to manage dynamic regions. Such regions enclose sets of feature points randomly sampled so as to retrieve motion and structure. Along the processing sliding window, tracked regions are propagated until reaching dynamic scene motion segmentation convergence.

**Figure 1:**
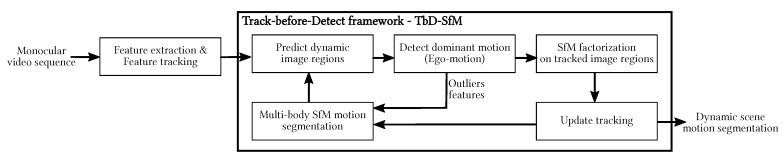
Motion segmentation with tracking objects.

Figure 1 illustrates the outline of the proposed approach, referred as TbD-SfM. In the following the sequential process is detailed.

### 4.1. Representation of Dynamic Regions

A dynamic region is represented by a horizontally oriented box with centroid coordinates (u,v), width, *w*, and height, *h* in pixels. In this context, dynamic regions enclose objects entities and associate theirs feature points along the sliding window. It is worth noting that ego-motion features cannot be correctly enclosed by a unique dynamic region. For this reason, this set of features is put aside from the tracking scheme. Only the remaining dynamic regions are then considered as potential dynamic objects.

### 4.2. Initialization

The TbD-SfM is initialized with rough motion segments (see Section 3 or alternatively [35]. In this stage, feature points are assigned to the inputted dynamic regions. Ego-motion is inferred as the dynamic region is composed of the larger set of feature points (dominant motion assumption). At this stage, a first estimation of their size and location is carried out on the set of dynamic regions.

### 4.3. Scene Analysis

Scene analysis starts by identifying features belonging to the dominant motion set, denoted as $W_{1}$. To this end, feature points enclosed in the dynamic regions $\left(W{}_{2p,\ldots ,np}\right)$ are removed from the trajectory matrix. The remaining features follow the dominant trajectory matrix:

(11) $$W_{1p}=\hat{W}-\left[W_{2p}|W_{3p}\left|\ldots \right|W_{np}\right]$$

where, $W_{np}$ represents the trajectory matrix of the *n*th motion. It is important to note that the set of features $W_{1p}$ can include missed classified features. A robust RANSAC-based motion estimation is carried out on the set $W_{1p}$ following the steps described in Section 3.2. The estimation of the dominant motion must fulfill a consensus set of features composed of at least *m* features. The consensus value, *m*, is determined by the minimum number of feature points (k) required to instantiate a motion estimate. That corresponds to the number of columns in $W_{np}$ as follows:

(12) $$m=col(W{}_{np})-k$$

The solution with the largest consensus among the set of features is selected. If there are multiple motion solutions with the same consensus, the one with the smallest mean reprojection error is maintained. In presence of multiple observed motions included in the set of features $W_{1p}$, the motion estimates might not achieve the minimum required consensus. This situation occurs when the number of outliers is greater than *k* feature points or when there is at least one new moving object in the scene. Motion factorization is applied to the set of unsegmented features in order to find new moving objects or to discard such features as outliers. The results of this stage are $W_{1}$, the structure ${\tilde{S}}_{1}$ and motion ${\tilde{M}}_{1}$ of the dominant motion, and the $W_{n}$, its structure ${\tilde{S}}_{n}$ and motion ${\tilde{M}}_{n}$ of the new objects that entered in the scene.

### 4.4. Motion Factorization on Dynamic Regions

The motions are factorized relying on the segmented feature points inside of each dynamic region $\left[W_{2p}|W_{3p}\left|\ldots \right|W_{np}\right]$. In each matrix it is assumed the presence of feature points following the *n*th moving object and outliers. Features classified as outliers by the motion factorization, are associated to other dynamic regions or finally discarded following their reprojection error. At this stage, feature points are classified in the trajectory matrix $\left[W_{2}|W_{3}\left|\ldots \right|W_{n}\right]$ and theirs structures and motions are recovered.

### 4.5. Number of Hypotheses

The number of motion hypothesis during RANSAC can be fixed assuming a known proportion of outliers on the dynamic region that should not be exceeded. The outlier proportion can be adaptive as presented in [34]. The number of motion hypotheses are computed with a probability of $p_{r}=99\%$ and k=8, as stated in the Equation (10).

### 4.6. Filtering

A bank of Kalman filters (KF) is implemented to manage and to infer the most probable states of the dynamic regions. Assuming that the observed moving objects in the sequence are subject to physical dynamics, these are expected to perform smooth changes in the image sequence. The state of a dynamic region in the image plane is tracked by a 8D vector. The track state is denoted by $x_{f}$ (see Equation (13)) consisting of the image centroid coordinates, $\left(x_{c},y_{c}\right)$, in pixel, the width, *w* and height, *h*:

(13) $$x_{f|f}={\left[x_{c},y_{c},w,h,v_{x},v_{y},\delta_{w},\delta_{h}\right]}^{T}$$

The state vector of the image region attributes also includes their first derivatives respectively ($v_{x},v_{y},\delta_{w},\delta_{h}$). Since an inter-frame linear and uniform motion is assumed, a linear Gaussian model is well suited for tracking purpose as is stated in Equation (14):

(14) $$\left\{\begin{array}{cc} x_{f}=A\cdot x_{f-1}+{\mathrm{ff}}_{f} & \alpha_{f}∼N\left(\alpha_{f};0,\Lambda_{f}\right)\\ y_{f}=C\cdot x_{f}+{\mathrm{fi}}_{f} & \beta_{f}∼N\left(\beta_{f};0,\Gamma_{f}\right) \end{array}\right.$$

where *A* and *C* represent the transition and the observation models, respectively. $x_{f-1}$ stands for the state vector in a previous sample frame and $y_{f}$ the multivariate observations. ${\mathrm{ff}}_{f}$ and ${\mathrm{fi}}_{f}$ are the state and observation noise following a zero-centered normal distribution with known variances.

#### 4.6.1. Track-to-Motion Association

Tracked regions states are predicted by means of its associated Kalman filter. State predictions enclose the set of points employed for motion factorization as illustrated in Figure 1. The features following the factorized motion update the tracked region if it satisfies a geometric distance criterion. The criterion correlates the tracked dynamic region and the region enclosing the detected factorized motion regarding their appearance and uncertainty-weighted state given by the inverse of the mean point reprojection error.

#### 4.6.2. Track Creation and Deletion

A dynamic region has to be detected in at least 60% of frames of the sliding window size so as provide enough evidence to initialize a filter to track it. The non-updated tracks are destroyed if theirs predictions are not reliable enough to be associated to new detected motions (i.e., 60% detection rate). A new moving object is detected using the points classified as outliers. The factorization method is applied over these feature points in order to find a new group that satisfies the reprojection error criterion $\epsilon_{hyp}$, see Section 3.

Hereafter, the outline of Tdb-Sfm is presented in Algorithm 2:

**Algorithm 2** Proposed Algorithm Framework
1: **procedure**
Framework(*W*)2:  **for**
frame=1 to last frame
**do**3:   **if**
frame==1
**then**4:    Motion Segmentation with baseline method Algorithm 15:    Get the dynamic objects positions6:   **else**7:    **if**
frame=≤F
**then**8:     Remove from *W* feature points belonging to dynamic objects9:     Find the Ego-motion feature points10:     Find the dynamic feature points11:     Search new motions in the outliers feature points12:     Feed the KF with the position of the dynamic objects13:    **else**14:     Predict positions and sizes of the objects15:     Remove the points in the motion objects areas16:     Find the Ego-motion feature points17:     Find the dynamic feature points18:     Search new motions in the outliers feature points19:     Update the position of the dynamic objects20:     Feed the KF with the position of the dynamic objects21:    **end if**22:    **end if**23:   **end if**24:   **end if**25:  **end for**26:  **end for**27:  **return**
$W_{n},M_{n},S_{n}$▹ Trajectory matrix segmented, Motion and Structure28: **end procedure**


## 5. Results

The baseline algorithm (Section 3) and TbD-SfM algorithm (Section 4) are evaluated in different urban scenarios using the Hopkins 155 (http://www.vision.jhu.edu/data/hopkins155/) and KITTI (http://www.cvlibs.net/datasets/kitti/) datasets. The Hopkins 155 dataset provides a sequence of images with small inter-frame motions. The images were recorded with a hand-held camera. The dataset provides the optical flow without tracking errors in the differences sequences. The 2D feature points are tracked along of sequences composed of 640 × 480 images acquired with a rate of 15 frames per second. KITTI dataset [36] has scenarios with greater dynamic complexity in comparison with Hopkins dataset. KITTI has 1392 × 512 images sampled in uncontrolled illumination conditions from a camera embedded on a moving car. The speed of the camera can reach 60 Km/h in some scenes. The dataset does not furnish the feature points in the scenes. This allows the possibility of tracking errors in the optical flow. Feature points are acquired by means of the Libviso2 extractor [37]. The scenes are processed in a temporal sliding window of 5-frames of size (Γ). The results obtained per sliding window are processed and the mean value is reported as a frame result. At least, 8 feature points are required for motion detection. The initialization of the TbD-SfM method is done with the baseline algorithm and the result is reported in the first frame. The values of ζ and ρ are selected heuristically and were set to ζ=1, ρ=0.07 in the experiments. Threshold values $\epsilon_{hyp}=\epsilon_{k}\cdot k\cdot \Gamma$ and $\epsilon_{pto}=\epsilon_{p}\cdot \Gamma$ are selected based on the performance of the method estimated with the confusion matrix, Table 1. The evaluation of the methods are done following: the reprojection error, the segmentation error and the outliers ratio.

The reprojection error stands for the average difference between trajectory matrix, *W*, and its corresponding estimate, $\tilde{W}$ as follows:

(15) $$Rep.\;Error=\frac{\sum \left(W-\tilde{W}\right)}{Total\;\#\;of\;points}$$

The segmentation error is defined in [11] as the misclassification of a point between the objects observed in the scene. It is computed with the Equation (17) as:

(16) $$Seg.\;Error=100\frac{\#\;of\;misclassified\;points}{Total\;\#\;of\;points}$$

Outliers are defined as points that do not meet the reprojection error criterion established by the threshold $\epsilon_{p}$ included on the RANSAC scheme. The outliers ratio is then computed as:

(17) $$Outliers\;Ratio=100\frac{\#\;of\;unclassified\;points}{Total\;\#\;of\;points}$$

### 5.1. Experimental Evaluation of Baseline Method

**Figure 2:**
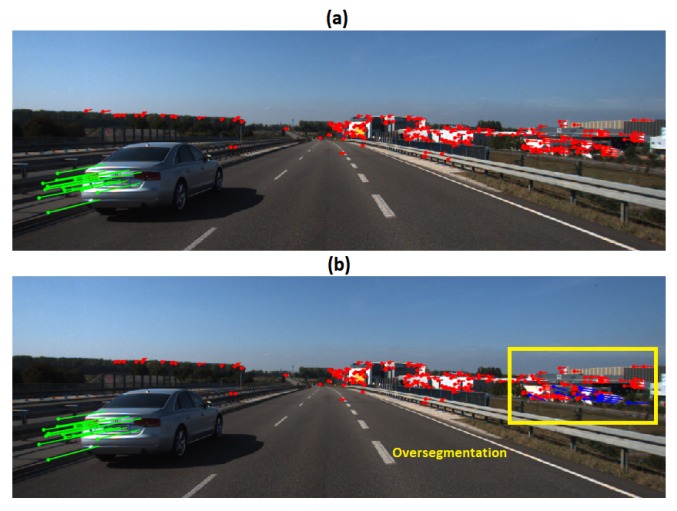
Results of Scene 1: (**a**) Motion trajectories; (**b**) Oversegmentation example.

The baseline algorithm was tested on the KITTI scenes road-2011_10_03_drive_0042 (Scene 1) and residential-2011_09_30_drive_0034 (Scene 2), the results were compared with [7]. Scene 1 involves two cars at high speed (around 55 km/h), the moving camera and a car passing from back to the front. 5 frames were processed, each one composed of 218 feature points. The Figure 2a illustrates the feature points trajectories of the dominant motion in red and the moving object in green. The Figure 2b exhibits an example of over-segmented motion obtained with $\epsilon_{k}=0.25$ pixels and $\epsilon_{p}=3$ pixels. Table 2 exhibits precision and recall results. For these tests 200 scene motion segmentation hypotheses were generated with the values of $\epsilon_{k}=0.3$ pixels and $\epsilon_{p}=4$ pixels. In the obtained results the moving object was correctly segmented, however, the dominant motion was divided into two motions. It is worth noting that even if there is no classification errors on moving objects, the set of dominant motion features might be, in some cases, over-segmented.

**Figure 3:**
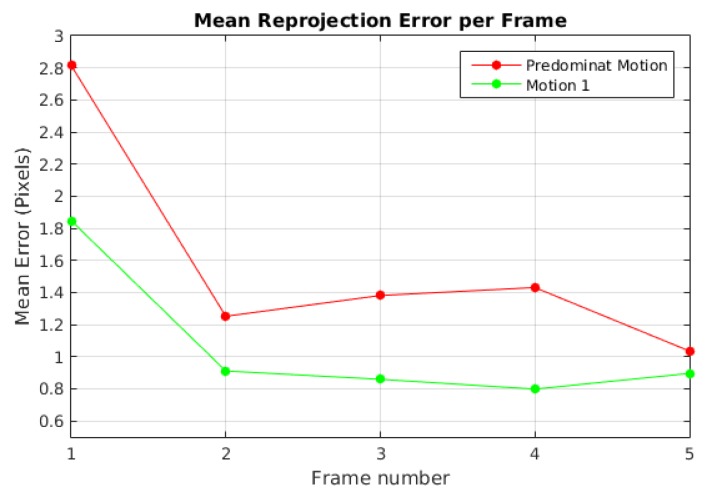
Mean reprojection error by frame for Scene 1.

Figure 3 displays the highest reprojection error of the baseline method in Scene 1 for the dominant motion and moving object with values of 2.8 pixels and 1.8 pixels, respectively.

**Figure 4:**
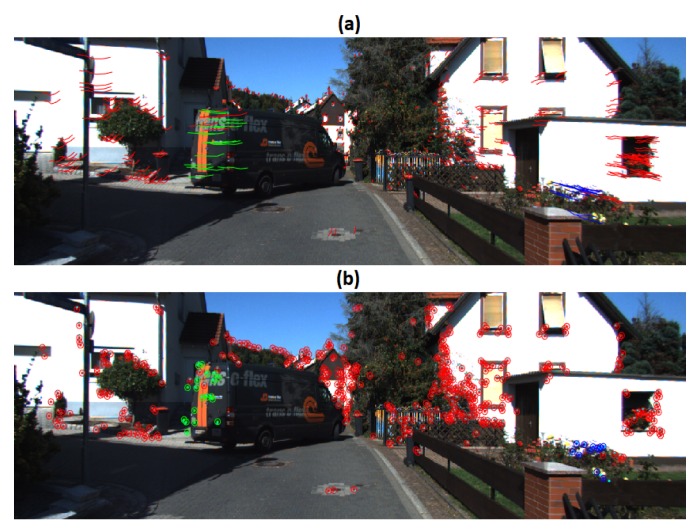
Results of Scene 2: (**a**) Trajectories of the vehicle; (**b**) Reprojection error.

In Scene 2, it is observed a vehicle moving in reverse direction and turning. The parameter values employed in this sequence were $\epsilon_{k}=0.25$ pixels and $\epsilon_{p}=3$ pixels. In Figure 4, three motions groups were detected: the dominant motion, the moving object and a group of 11 feature points. The observed over-segmentation can be coped with a fine tuning of the $\epsilon_{p}$ threshold.

Table 3 summarizes the results obtained for Scene 1 and 2 and includes the performances reported in the state of the art [7]. In Scene 1, the baseline method achieves a motion segmentation error of 0% with a mean and median reprojection error lower than the ones reported on [7]. However, in the Scene 2 the segmentation error was greater with 3.3% and the mean and median reprojection error were lower than the ones of [7]. These results let us assume that our implementation is reliable enough for a fair comparison.

### 5.2. Experimental Evaluation of TbD-SfM

The baseline and TbD-SfM methods were tested and compared. Hereafter, a first set of experiments using Hopkins 155 traffic dataset is reported. It is recalled that TbD-SfM uses the results provided by the baseline method in the first frame as an initial knowledge of the scene (rough segmentation). TbD-SfM is able to detect and to segment moving objects present in the scene as well as new objects that may appear or leave.

**Figure 5:**
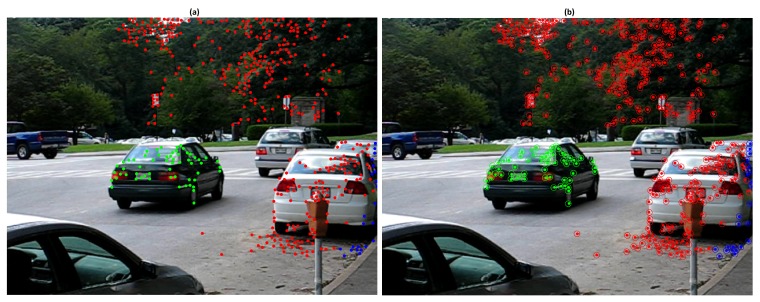
Baseline method results for Scene 3: (**a**) First frame segmentation; (**b**) Reprojection error.

Figure 5 presents a scene composed of two simultaneous motions called Car2 (named Scene 3). The baseline method was parametrized considering 200 scene motion segmentation hypotheses by frame along the sequences. Thirty frames were processed using 26 sliding windows, each frame includes 490 feature points. The best precision and recall values were obtained with $\epsilon_{k}=0.5$ pixels and $\epsilon_{p}=1$ as reported in the Table 4.

In Figure 5, the segmentation and the reprojection error obtained with the baseline method in the first frame of the scene are shown. The moving object was segmented correctly, however, the dominant motion was over-segmented. A third group in blue was created with few feature points. The right image exposes the reprojection error in the first frame.

**Figure 6:**
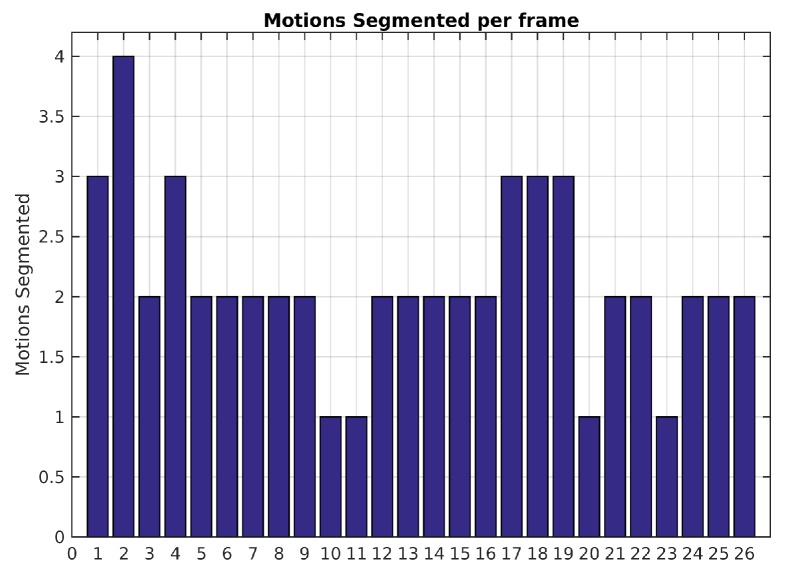
Number of motions by frame with baseline method.

Figure 6 shows the number of segmented motions reported by the baseline method along the sequence. Since the scene is only composed of two independent motions, results with more than 2 are over-segmented and less than 2 are under-segmented. The low recall value of 0.66 (see Table 4) is caused by the incorrect segmentation in the frames 10, 11, 20 and 23. This is probably due to the fact that the observed vehicle slows down. Decreasing the value of $\epsilon_{p}$ may help to segment small inter-frame motions but can also lead to over-segmented scenes.

**Figure 7:**
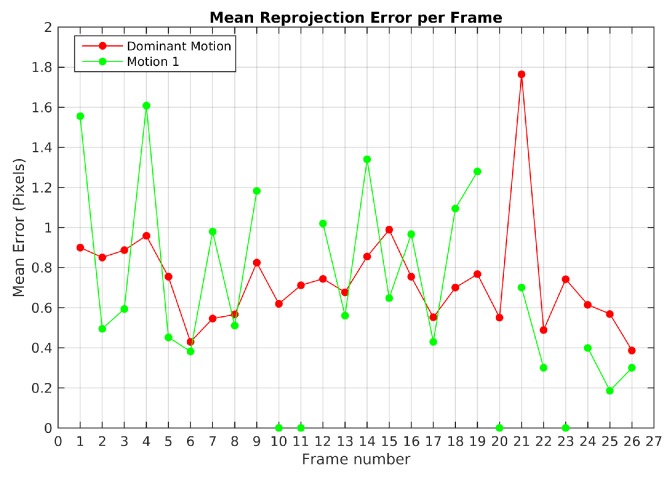
Mean reprojection error of detected motions for Scene 3 with baseline method.

Figure 7 plots the mean reprojection error of the motions detected by the baseline method in Scene 3. In green dot-line, the moving object motion and in red dot-line the dominant motion (ego). Since the moving object was missed and its feature points were assigned to the dominant motion set in frames 10, 11, 20 and 23, no reprojection error was computed. The highest reprojection error was 1.9 pixels in frame 21 for the dominant motion and 1.6 pixels in the frame 4 for the moving object motion. Despite these reprojection errors, motions were segmented correctly.

The TbD-SfM was parametrized assuming an outlier ratio of 30% and thresholds values $\epsilon_{k}=0.5$ pixels and $\epsilon_{p}=3$. Threshold values were selected following precision and recall scores computed for the first frame of Scene 3 and reported in Table 5. A good feature points classification was obtained and no over-segmented areas were observed in the frame.

**Figure 8:**
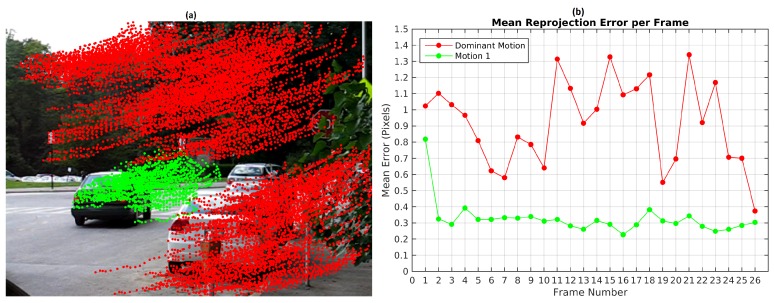
TbD-SfM results for Scene 3: (**a**) Motions segmented; (**b**) Mean reprojection error evolution.

For the complete sequence Scene 3, the two motions were segmented correctly using TbD-SfM. The highest mean reprojection error was of 1.35 pixels for the dominant motion and 0.8 pixels for the moving object as shown in Figure 8.

**Figure 9:**
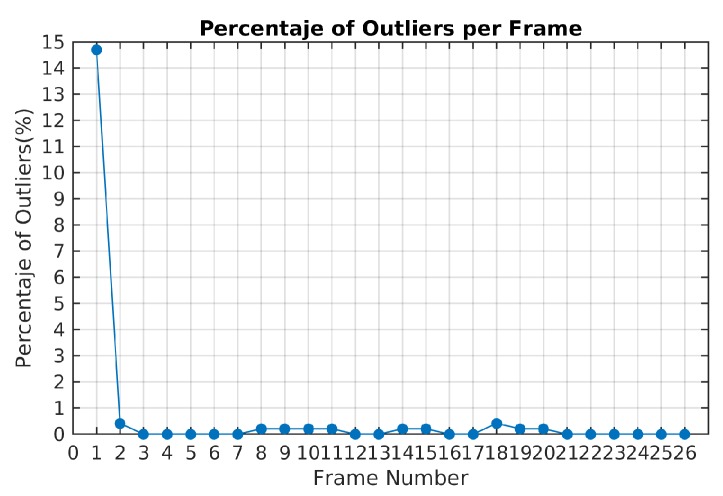
Ratio of outliers using TbD-SfM method for Scene 3.

The ratio of outliers per frame is illustrated in Figure 9. The highest value corresponds to the first frame estimation. In the next frames, the ratio of outliers with the TbD-SfM approach was less than 1%.

**Figure 10:**
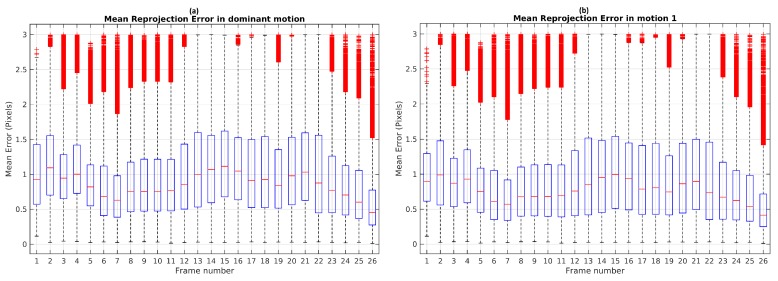
Mean reprojection error for Scene 3: (**a**) Dominant motion; (**b**) Dynamic object.

A Monte Carlo experiment was carried out in order to evaluate the repeatability and the stability of TbD-SfM results. To this end, scene segmentation was performed on 100 repetitions. The highest reprojection error was limited by the threshold $\epsilon_{p}=3$. The boxplot illustrates (see Figure 10) that frames 13, 14, 15, 18, 21 and 22 used the range established in $\epsilon_{p}$. Others frames had the maximum boxplot value of the mean reprojection error results less than $\epsilon_{p}$ threshold.

**Figure 11:**
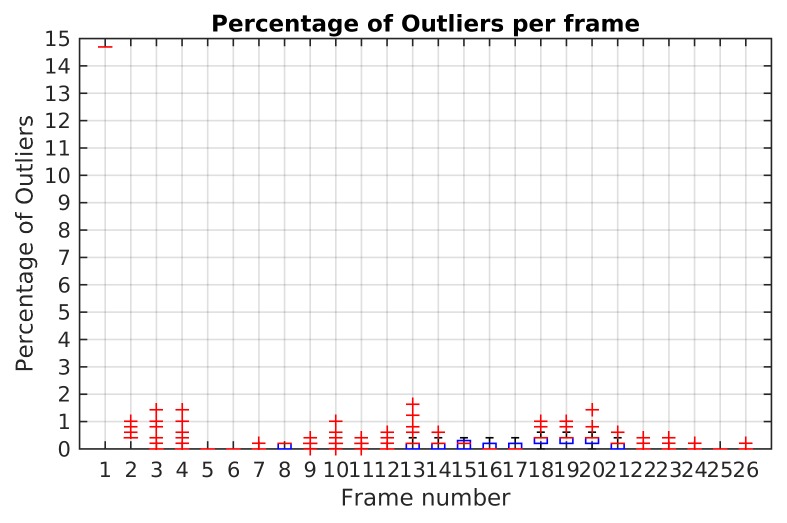
Outliers percentage in Monte-Carlo experiment for Scene 3 using TbD-SfM.

The highest percentage of outliers observed along Scene 3 is less than 2% as shown Figure 11. At least 98% of feature points by frame were correctly classified and not rejected as outliers.

**Figure 12:**
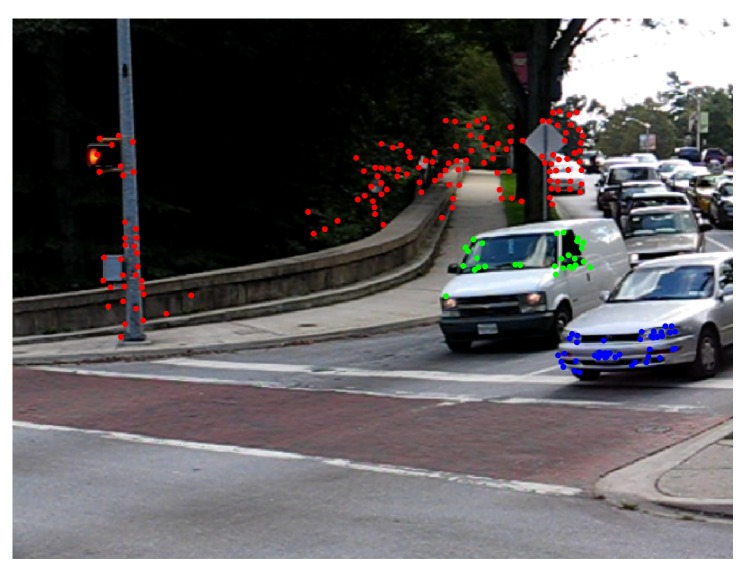
First frame segmentation for Scene 4.

Figure 12 shows motion segmentation for the first frame of Hopkins 155 Car 9 sequence called Scene 4. The scene is composed of three simultaneous independent motions: the dominant motion (static objects in red) and two moving objects (green and blue). This sequence is a challenging use case since the observed objects moves at slow speed. Twenty four frames were processed with 220 feature points per frame. The baseline method was set to consider 300 scene motion segmentation hypotheses by frame. Results of the sequence are quantified in Table 6. Reported results were obtained with threshold values $\epsilon_{k}=0.25$ pixels and $\epsilon_{p}=2.5$ pixels. The Figure 12 illustrates the motion segmentation result for the first frame.

**Figure 13:**
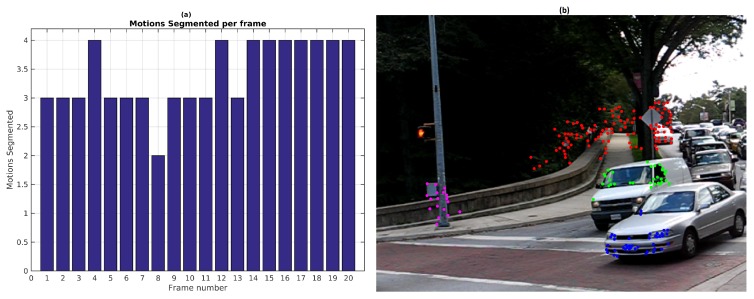
Baseline method results for Scene 4: (**a**) Number of motions by frame; (**b**) Motion segmentation.

Despite the fact that precision and recall scores in Table 6 are high, motion segmentation errors are still present along the sequence. That is the case for frames 4, 12 and from 14 to 20 where the baseline method over-segments motions and misses one of them in frame 8 (Figure 13). Figure 13b illustrates as an example the segmentation result of frame 12.

**Figure 14:**
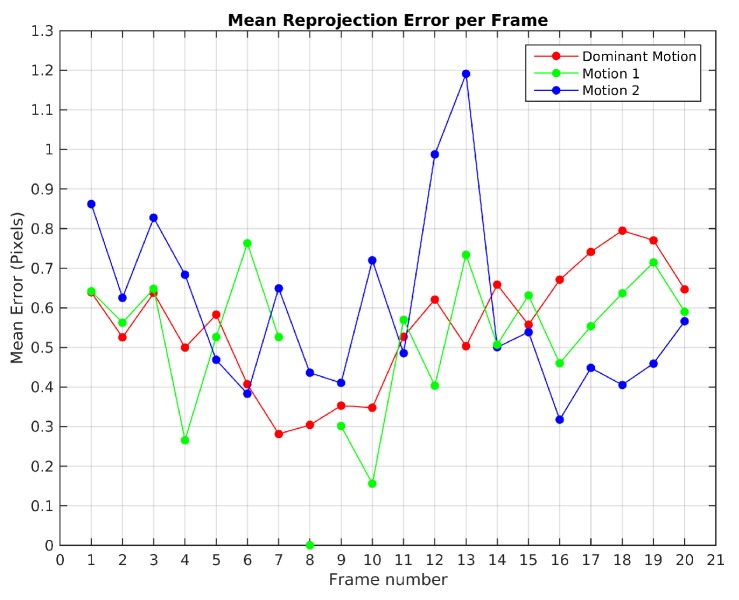
Mean reprojection error for Scene 4 with baseline method.

Figure 14 illustrates the mean reprojection error evolution in Scene 4, the highest value was obtained in the 13th frame for the 2nd observed motion with 1.2 pixels. The 8th frame shows that the 1st observed motion was not detected.

**Figure 15:**
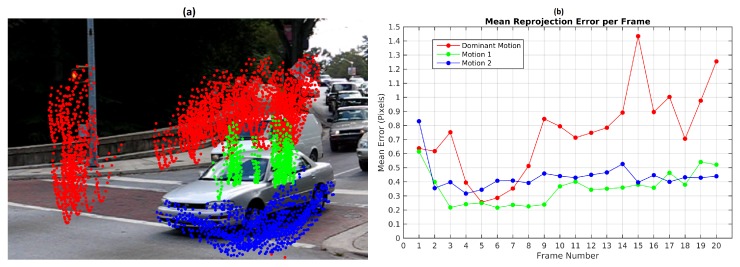
TbD-SfM results for Scene 4: (**a**) Motions segmented; (**b**) Mean reprojection error.

The TbD-SfM was tested with the same values $\epsilon_{k}=0.25$ pixels and $\epsilon_{p}=2.5$ and a RANSAC outlier ratio of 30%. The three motions were segmented correctly. Figure 15b shows the mean reprojection error with a highest error of 1.45 pixels for the dominant motion. The highest reprojection error in the moving objects were less than 0.55 pixels.

**Figure 16:**
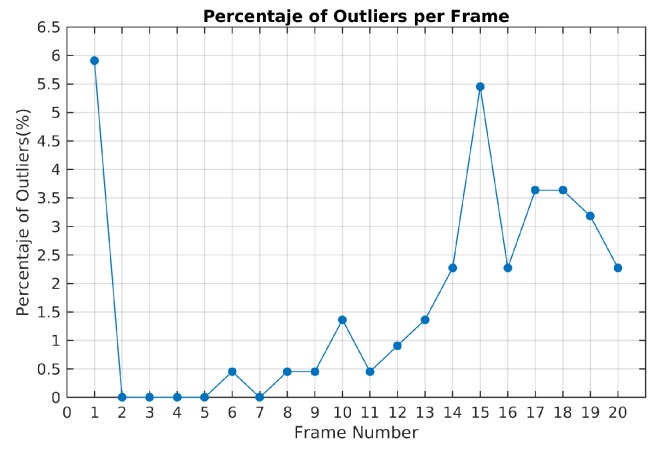
Ratio of outliers with TbD-SfM method for Scene 4.

The highest percentage of outliers was obtained in frame 15 as illustrated in Figure 16. In this frame, it was also obtained the highest reprojection error in the dominant motion. In this case, the selected hypotheses increases the reprojection error in the feature points and some of them were rejected. A high percentage of outliers are coming from the dominant motion even when the reprojection error is less than 1.5 pixels. The opposite situation was presented in the frames 2, 3, 4 and 5 where all the feature points were segmented correctly.

**Figure 17:**
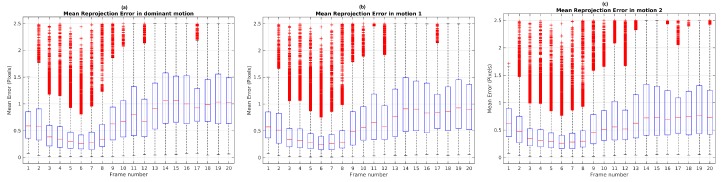
Mean reprojection error results of Monte Carlo test: (**a**) Dominant motion; (**b**) Motion 1. (**c**) Motion 2.

The results of the Monte Carlo experiment with TbD-SfM in Scene 9 are shown in Figure 17. The highest reprojection error was limited by the threshold $\epsilon_{p}=2.5$. In a scene composed of three observed motions, the frame range from 3 to 8 shows that the maximum boxplot value for the mean reprojection error is less than 1 pixel. After frame 10, the upper whisker is greater because the moving objects are getting closer to the camera.

**Figure 18:**
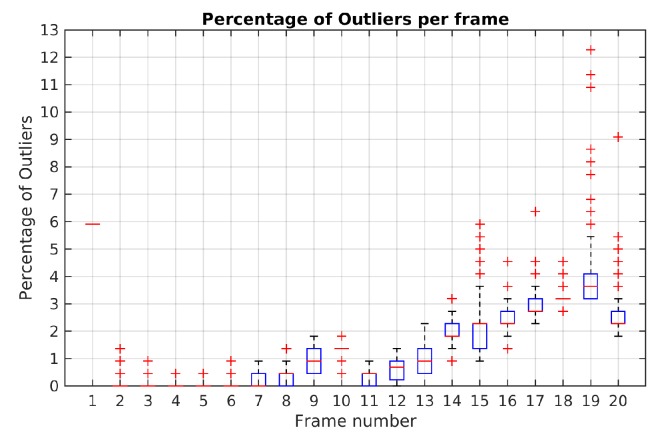
Outliers percentage in Monte-Carlo experiment for Scene 4 using TbD-SfM.

Figure 18 illustrates the boxplot results of Monte-Carlo experiment in Scene 4. It is noted that until frame 14 the maximum percentage of outliers obtained was 3.1%. In frame 19, it is shows a maximum boxplot value of 5.5% and the highest percentage of outliers with 12.2%. Except for this frame, the maximum boxplot value for the percentage of outliers is less than 4%.

Table 7 summarizes the evaluation results of the Monte Carlo experiments in Scene 3 and Scene 4 using TbD-SfM method. In Scene 3, TbD-SfM achieved a mean reprojeccion error of 1.25 pixel, a segmentation error of 0.01% and a mean outliers percentage of 0.8%. In Scene 4, it was obtained a mean reprojeccion error of 0.84 pixel, a segmentation error of 0.19% and a mean outliers percentage of 3.1%.

**Figure 19:**
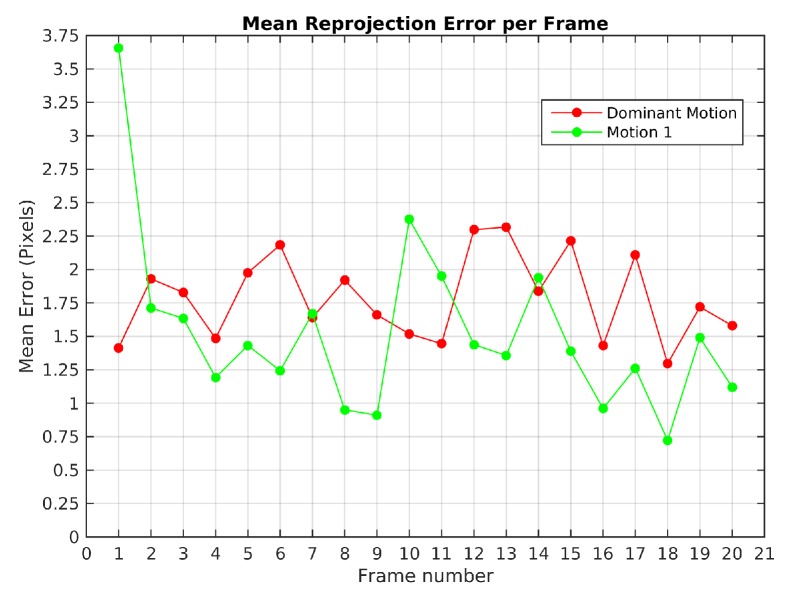
Mean reprojection error in Scene 1 with baseline method.

Scene 1 (Figure 2) from KITTI dataset was processed with the baseline algorithm and TbD-SfM. A sequence of 20 frames with an average of 185 feature points by frame was processed. The baseline method was used to create 200 scene motion segmentation hypotheses by frame with the values $\epsilon_{k}=0.875$ and $\epsilon_{p}=3$. The Figure 19 illustrates the mean error reprojection error for the two segmented motions, the highest value was 3.6 pixels for the moving object in the first frame.

**Figure 20:**
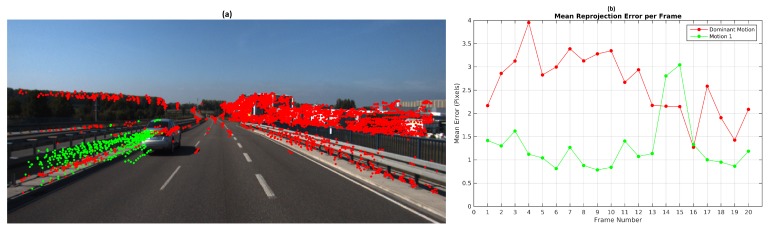
TbD-SfM results for Scene 1: (**a**) Motion segmentation; (**b**) Mean reprojection error.

TbD-SfM was set to assume an outliers ratio of 35%. The highest mean reprojection error was in the 4th frame of the dominant motion with 4 pixels as shown in Figure 20b. One can notice that reprojection errors in KITTI dataset are higher than the ones achieved on Hopkins. Since Hopkins provides error-free feature tracking, reprojeccion errors are greatly improved. KITTI experiments shows the robustness of the proposed method to feature tracking errors and their impact in terms of the reprojeccion error.

The segmentation results along the sequence are presented in Figure 20a. The feature points located in the side-view mirror of the vehicle were not segmented correctly. Since these points are observed in some frames outside of the predicted area, they were segmented in another group or classified as outliers. It was obtained a segmentation error of 1.4% along the sequence. The results are detailed in the Table 8.

**Figure 21:**
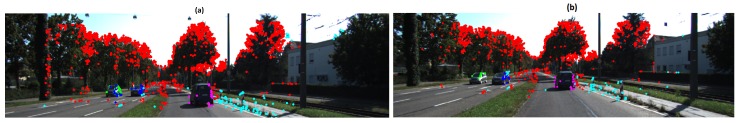
TbD-SfM results for Scene 5: (**a**) 1th frame segmentation; (**b**) 4th frame segmentation.

TbD-SfM efficiently addresses the scalability problem presented in the baseline method when the number of simultaneous motions increases. In the Scene 5, the scalability of TbD-SfM was tested in a scenario with 4 simultaneous motions as shown in Figure 21. There are two moving objects approaching to camera with different speeds and a third one moving along the moving camera. 8 frames were processed with 4 sliding windows, an average of 1450 feature points are observed by frame. The first frame segmentation was obtained with the baseline method considering 400 scene motion segmentation hypotheses with the parameters of $\epsilon_{k}=0.25$ pixels and $\epsilon_{p}=3$. In the first frame, some segmentation errors were observed: some feature points of the moving object 1 (green) were assigned to the moving object 2 (blue). However, the TbD-SfM procedure allowed to correct these errors and enhanced the segmentation as shown the frame 4. The outliers feature points are shown in cyan color.

**Figure 22:**
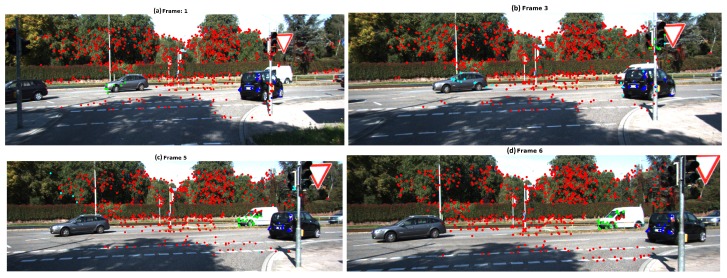
TbD-SfM results for Scene 6: (**a**) Frame 1; (**b**) Frame 3; (**c**) Frame 5; (**d**) Frame 6.

In Scene 6, TbD-SfM was implemented in a sequence under particular characteristics. The moving camera is turning right, objects enter or leave the scene and some of them are partially occluded. This scene allows to test the detection and segmentation of new moving objects. It were processed 6 frames with an average of 670 feature points per frame. The moving objects are represented by the green and blue feature points. The parameter values employed in this sequence were $\epsilon_{k}=0.75$ pixels, $\epsilon_{p}=3.5$ and it was assumed an outliers ratio of 45%. Figure 22 illustrates the results obtained by frame with TbD-SfM approach. In the first frame, it was detected 3 simultaneous motions. The green moving object has a partial occlusion by the ego-motion feature points located over the traffic light post. In the third frame, a small group of feature points was segmented as other moving object over the traffic light post, however, this group is not detected in the next frames. The outliers feature points are represented in cyan, this points over the gray car were not associated because they do not meet the reprojection error criterion $\left(\epsilon_{hyp}\right)$. Some feature points of a new object(white car) were segmented with the dominant motion. In the 5th frame, the white car was detected as new moving object for first time and some segmentation errors. In the 6th frame the new moving object is detected with a better segmentation. The results evaluated are reported in the Table 8.

**Figure 23:**
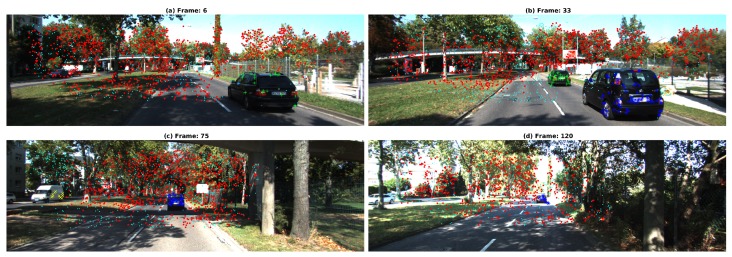
TbD-SfM results for Scene 7. The motions segmented are indicated by colors and markers. Red points represent the ego-motion. The 1st, 2nd and 3rd motion are represented by the green plus signs, blue asterisk and yellow cross respectively.

It is worth to mention that performances and execution time of the TbD-SfM were also evaluated on a long sequence context. In the Scene 7, 130 frames were processed involving a moving ego-camera, two cars passing from back to the front and a third car approaching. As an example, the Figure 23a shows the 6th frame where the first moving object was segmented. A second car was then detected and segmented as shown the Figure 23b. Figure 23c presents the second car marked in blue is occluding the first detected moving object. At the left side, a van approaching to the ego-camera that was segmented and marked in yellow. Finally, the Figure 23d illustrates 120th frame where the object was segmented while it moves away. Performance results are reported in the Table 8.

**Figure 24:**
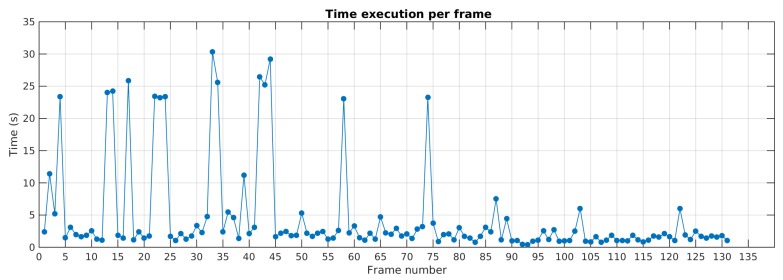
Execution time along the sequence for Scene 7.

The Figure 24 details the execution time per frame along the sequence in Scene 7. The results show that before the 50th frame, run time is higher due to a greater amount of dynamic feature points. After, run time decreases. It is worth noting that the detection of new motions requires more processing time due to feature resampling task. That can be observed by run time peaks in frames 14, 22, 34, 44, 57, 74. This processing time can be greatly enhanced by parallelizing or pipelining feature resampling and motion tracking threads.

TbD-SfM was tested in car sequences of Hopkins dataset for allowing comparison with other methods. The dataset has 8 scenes with two simultaneous motions and 3 scenes with three simultaneous motions. The algorithm was run once by sequence and the results reported in the Table 9. The highest mean reprojection error was of 1.25 pixels for Car2 sequence and the highest segmentation error and outliers percentage were 0.2% and 6.1%, respectively, for the Truck2 sequence.

Table 10 shows a benchmark comparison of the car sequences results using TbD-SfM (Table 9) and other state-of-the-art methods [38,39]. The results presented in the Table 10 shows that TbD-SfM achieves a lower segmentation error in scenes with two and three simultaneous motions in comparison to methods presented in [18,20,21,22,24,26,27,28,29,30,31,32]. TbD-SfM obtains a segmentation error of 0.07% for sequences involving three simultaneous motions. This error is higher in comparison to HSIT [23] that reaches a perfect segmentation. In contrast, the segmentation error in two simultaneous motions sequences of TbD-SfM is 0.02% compared to 1.65% of HSIT that is 82 times lower. TbD-SfM has similar performance in comparison with the DCT [19]. The DCT segmentation error was 0.05% considering all the sequences of the dataset, while TbD-SfM segmentation error was lower in datasets with two motions by a difference of 0.03% and higher by 0.02% for three motions dataset. Comparing TbD-SfM to the baseline method, the segmentation error is higher by a difference of 0.02% in sequences with two simultaneous motions and lower by 0.04% in datasets with three simultaneous motions. In particular, TbD-SfM have obtained a greater number of feature points correctly segmented in comparison with the baseline method as shown the percentage of outliers in the Table 9. It is worth noting TbD-SfM achieves a denser feature segmentation than the baseline approach. That is because the baseline approach performs an optimization step intended to enhance motion segmentation by rejecting feature points with a high reprojection error. This procedure can certainly improve motion estimates but it also reduces the number of feature points that represent a motion. Objects with few features may be easily lost or missed detected.

The results show that our algorithm outperforms the RANSAC formulation proposed in [31]. The reprojection error obtained with TbD-SfM algorithm can be reduced with an optimization method over the RANSAC formulation as described in [40,41].

The reported experiments were obtained thanks to Matlab implementations on a laptop with processor i-7 2.6 GHz and 16 GB-RAM. The average running time of the baseline method for two, three and four simultaneous motions were 85.2 s, 259 s and 6360 s. For TbD-SfM method execution time decreases in average to 3.5 s, 3.9 s and 78.3 s for two, three and four simultaneous motions, respectively.

## 6. Conclusions

This paper proposed an efficient TbD-SfM framework able to infer independent motions (euro-motions) and ego-camera trajectory under a 6-DOF motion model. Compared to complex existing motion segmentation approaches, the proposed methodology represents a reliable vision-only alternative for sensors-based dynamic scene analysis and VSLAM applications. The implementation of the TbD-SfM in SfM allows us to drastically decrease the number of trial hypotheses required for a scene motion segmentation without the use of kinematics constraints. Thanks to this, our method is scalable and its advantages were thoroughly demonstrated in scenes with more than two simultaneous motions. The TbD-SfM constitutes a feasible motion segmentation algorithm for monocular vision systems with a bounded complexity intended to an embedded system implementation. A Hardware–Software co-design approach remains an issue to be addressed and constitutes a perspective of this work in order to achieve real-time performances. To this end, a further study of an embedded HPS (Hardware Processing System) based on a GPU or a FPGA architecture will be carried out in order to design a sensor implementing high-level on-chip pre-processing.

## Figures and Tables

**Table 1:** Confusion Matrix.

	Actual Classification		
Predictive classification		Yes	No
	Yes	True Positives (TP)	False Positives (FP)
	No	False Negatives (FN)	True Negatives (TN)

**Table 2:** Precision(P) vs. Recall(R) of Baseline method for Scene 1.

$\epsilon_{k}\;⟶$	0.3		0.4		0.5	
$\epsilon_{p}\;↓$	P	R	P	R	P	R
1	0.83	1	1	1	1	0.46
2	1	1	1	1	1	1
3	1	1	1	1	1	1
4	1	1	1	1	1	1
5	0.94	1	1	1	1	1

**Table 3:** Reprojection and segmentation errors obtained for Scene 1 and Scene 2.

	Sequence	Number of Frames	Number of Points	Mean Reprojection Error (pixels)	Median Reprojection Error (pixels)	Segmentation Error (%)
Reported in [7]	Scene 1	5	193	1.63	1.43	0
Baseline	Scene 1	5	218	1.54	1.18	0
Reported in [7]	Scene 2	5	573	2.14	1.67	1.57
Baseline	Scene 2	5	477	1.8	1.28	3.35

**Table 4:** Precision and Recall values for sequence of Scene 3.

$\epsilon_{k}\;⟶$	0.25		0.5		0.75		1	
$\epsilon_{p}\;↓$	P	R	P	R	P	R	P	R
0.75	0.88	0.58	0.74	0.63	0.79	0.69	0.94	0.58
1	0.80	0.72	0.94	0.66	0.51	0.68	0.7	0.78
1.5	0.37	0.77	0.4	0.8	0.53	0.7	0.4	0.7
2	0.35	0.78	0.27	0.82	0.34	0.75	0.43	0.67
3	0.17	0.90	0.16	0.85	0.18	0.86	0.18	0.82

**Table 5:** Precision and Recall scores for threshold selection in the 1st frame of Scene 3.

$\epsilon_{k}\;⟶$	0.25		0.5		0.75		1		1.25	
$\epsilon_{p}\;↓$	P	R	P	R	P	R	P	R	P	R
2	1	0.93	1	0.93	1	0.5	1	0.93	1	0.75
3	1	0.93	1	0.93	1	0.88	1	0.83	1	0.92
4	1	0.77	1	0.89	1	0.93	0.98	0.89	1	0.93

**Table 6:** Precision and Recall scores in Scene 4 using baseline method.

$\epsilon_{k}\;⟶$	0.125		0.25		0.375		0.5	
$\epsilon_{p}\;↓$	P	R	P	R	P	R	P	R
0.5	0.99	0.56	0.99	0.65	0.95	0.73	0.94	0.8
1	0.99	0.88	0.99	0.83	0.89	0.79	0.99	0.81
1.5	0.98	0.91	0.94	0.87	0.91	0.82	0.93	0.92
2	0.86	0.93	0.99	0.95	1	0.93	0.91	0.87
2.5	1	0.92	1	0.96	1	0.94	1	0.88
3	1	0.82	0.74	0.83	0.8	0.79	0.68	0.88

**Table 7:** Results with TbD-SfM method in Monte-Carlo experiment for Scene 3 and Scene 4.

Sequence	Number of Frames	Number of Points	Mean Reprojection Error (pixels)	Median Reprojection Error (pixels)	Segmentation Error (%)	Mean Outliers Percentage (%)
Car2	26	490	1.25	0.94	0.015	0.8
Car9	20	220	0.84	0.58	0.19	3.1

**Table 8:** Results reported for the KITTI datasets.

Sequence	Method	Number of Motions	Number of Frames	Number of Points	Mean Reprojection Error (pixels)	Median Reprojection Error (pixels)	Segmentation Error (%)	Mean Outliers Percentage (%)
Scene 1	Baseline	2	18	185	1.98	2.04	2.16	7.2
Scene 1	TbD-SfM	2	26	185	1.53	1.7	1.45	5.71
Scene 5	TbD-SfM	4	4	1450	1.22	1.15	0.24	3.22
Scene 6	TbD-SfM	2 and 3	6	670	1.37	1.24	1.5	1.5
Scene 7	TbD-SfM	3 and 4	130	1410	5.32	5.76	1.45	13.3

**Table 9:** TbD-SfM results for Hopkins dataset car sequences.

Sequence	Number of Motions	Number of Frames	Number of Points Per Frame	Mean Reprojection Error (pixels)	Median Reprojection Error (pixels)	Segmentation Error (%)	Mean Outliers Percentage (%)
Car1	2	16	307	1.10	0.96	0	1.09
Car2	2	26	490	1.25	0.93	0	0.73
Car3	3	13	548	0.97	0.79	0.07	3.85
Car4	2	50	147	0.78	0.52	0	2.3
Car5	3	30	391	0.47	0.29	0	0.1
Car6	2	27	464	0.44	0.35	0.03	0.1
Car7	2	21	502	0.88	0.75	0	0.1
Car8	2	21	192	0.74	0.58	0	0.37
Car9	3	20	220	0.65	0.47	0.15	1.75
Truck1	2	26	188	1	0.82	0	0.16
Truck2	2	18	331	1.07	0.94	0.2	6.1

**Table 10:** TbD-SfM results compared with other methods for Hopkins dataset car sequences.

Method	Reprojection Error (pixels)	Mean Segmentation Error for 2 Motions (%)	Median Segmentation Error for 2 Motions (%)	Mean Segmentation Error for 3 Motions (%)	Median Segmentation Error for 3 Motions (%)
Our TbD-SfM	0.85	0.02	0	0.07	0.07
Baseline [25]	0.091	0	0	0.11	0.24
MLBS [24]	-	8.86	-	25.1	-
HSIT [23]	-	1.65	-	0	-
IfSC [22]	-	1.25	-	3.97	-
MoGR [21]	-	1.24	-	4.97	-
RV [20]	-	0.44	-	1.88	-
DCT [19]	-	0.05	0	0.05	0
MSMC [18]	-	0.66	-	0.17	-
SLBF [26]	-	0.2	0	0.38	0
SSC [27]	-	1.2	0.32	0.52	0.28
GPCA [28]	-	1.41	0	19.83	19.55
ALC [29]	-	2.83	0.3	4.01	1.35
LLMC [12]	-	2.13	0	5.62	0
LSA [30]	-	5.43	1.48	25.07	23.79
RANSAC [31]	-	2.55	0.21	12.83	11.45
MSL [32]	-	2.23	0	1.8	0
